# Human Mas-Related G Protein-Coupled Receptors-X1 Induce Chemokine Receptor 2 Expression in Rat Dorsal Root Ganglia Neurons and Release of Chemokine Ligand 2 from the Human LAD-2 Mast Cell Line

**DOI:** 10.1371/journal.pone.0058756

**Published:** 2013-03-07

**Authors:** Hans Jürgen Solinski, Franziska Petermann, Kathrin Rothe, Ingrid Boekhoff, Thomas Gudermann, Andreas Breit

**Affiliations:** 1 Walther-Straub-Institut für Pharmakologie und Toxikologie, Ludwig-Maximilians-Universität München, Munich, Germany; 2 Department of Neurology, Klinikum rechts der Isar, Technical University Munich, Munich, Germany; Medical School of Hannover, United States of America

## Abstract

Primate-specific Mas-related G protein-coupled receptors-X1 (MRGPR-X1) are highly enriched in dorsal root ganglia (DRG) neurons and induce acute pain. Herein, we analyzed effects of MRGPR-X1 on serum response factors (SRF) or nuclear factors of activated T cells (NFAT), which control expression of various markers of chronic pain. Using HEK293, DRG neuron-derived F11 cells and cultured rat DRG neurons recombinantly expressing human MRGPR-X1, we found activation of a SRF reporter gene construct and induction of the early growth response protein-1 via extracellular signal-regulated kinases-1/2 known to play a significant role in the development of inflammatory pain. Furthermore, we observed MRGPR-X1-induced up-regulation of the chemokine receptor 2 (CCR2) via NFAT, which is considered as a key event in the onset of neuropathic pain and, so far, has not yet been described for any endogenous neuropeptide. Up-regulation of CCR2 is often associated with increased release of its endogenous agonist chemokine ligand 2 (CCL2). We also found MRGPR-X1-promoted release of CCL2 in a human connective tissue mast cell line endogenously expressing MRGPR-X1. Thus, we provide first evidence to suggest that MRGPR-X1 induce expression of chronic pain markers in DRG neurons and propose a so far unidentified signaling circuit that enhances chemokine signaling by acting on two distinct yet functionally co-operating cell types. Given the important role of chemokine signaling in pain chronification, we propose that interruption of this signaling circuit might be a promising new strategy to alleviate chemokine-promoted pain.

## Introduction

Primate-specific Mas-related G protein-coupled receptors-X1 (MRGPR-X1) have originally been described to be selectively expressed in small-diameter dorsal root ganglia (DRG) neurons [1], [2]. However, recently significant MRGPR-X1 mRNA levels were also detected in connective tissue mast cells (CTMC) and the leukaemia-derived human mast cell line (LAD)-2 [3], [4]. The endogenous agonist of MRGPR-X1, bovine adrenal medulla (BAM) peptide 8–22, is cleaved from pro-enkephalin, and several studies reported activation of the G_q_ pathway by MRGPR-X1 in over-expression systems [1], [5], [6], [7]. Studies from our laboratory revealed that MRGPR-X1 engage phospholipase-Cβ to release calcium form the endoplasmatic reticulum and activate the proalgetic transient receptor potential cation channel V1. In sharp contrast to most if not all G_q_-coupled receptors MRGPR-X1 do not undergo agonist-promoted endocytosis [6], [8]. In line with direct TRPV1 activation by MRGPR-X1 observed at the cellular level, application of BAM8-22 to healthy human volunteers provoked pain-like sensations pointing to acute nociceptive functions of MRGPR-X1 [9]. In contrast, over-expression of MRGPR-X1 in rat dorsal root ganglia (DRG) neurons resulted in BAM8-22-mediated inhibition of voltage-gated calcium currents via G_i/o_ proteins believed to blunt pain perception [10]. Thus, MRGPR-X1 play a significant role in acute human pain perception, but the underlying signaling pathways are still poorly defined. Furthermore, the impact of MRGPR-X1 on gene expression still remains largely elusive. This is of particular interest, because alterations in gene expression are often associated with chronic pain syndromes. In general, G protein-activating neuropeptides have been reported to affect gene expression via cAMP response elements (CRE) or serum response elements (SRE). CRE is activated by means of its interaction with the CRE binding protein (CREB) [11], whereas SRE activity is enhanced after binding to serum response factors (SRF) and to ternary complex factors (TCF) such as the E twenty-six-like transcription factor-1 (ELK-1) [12]. Interactions between CRE and CREB are enhanced after phosphorylation of the latter protein by numerous down-stream kinases of GPCR signaling such as protein kinase A or extracellular signal-regulated kinases-1/2 (ERK-1/2) [13]. Likewise, the affinity of the ELK-1/SRF/SRE complex is increased after phosphorylation of ELK-1 by ERK-1/2 [14]. Recent data also suggested a role for calcium/calcineurin-induced activation of nuclear factors of activated T cells (NFAT) in G protein-coupled receptor (GPCR)-promoted gene expression [15], [16]. Of note, CREB-, TCF/SRF- or NFAT-dependent gene expression is thought to induce maladaptive processes leading to neuronal dysfunction or pain chronification [16], [17], [18], [19], [20], [21].

Given the strong link between alterations in gene expression and pain chronification we herein analyzed effects of BAM8-22 on gene expression-regulating signaling pathways in previously reported human HEK293 or F11 (rat DRG neurons × murine NG18TG-2) cells stably expressing MRGPR-X1 and in cultured rat DRG neurons transiently expressing MRGPR-X1 [6], [8]. We observed that MRGPR-X1 induce gene expression in all three cell models tested and of particular interest that BAM8-22-induced expression of chemokine receptors 2 (CCR2), which has been linked to neuropathic pain syndromes [22], . In LAD-2 mast cells endogenously expressing MRGPR-X1, we detected significant release of the endogenous CCR2 agonist chemokine ligand 2 (CCL2) after BAM8-22 stimulation. Thus, we propose a MRGPR-X1-induced chemokine signaling circuit that involves induction of CCR2 expression in DRG neurons and CCL2 release in mast cells.

## Materials and Methods

### Materials and Plasmid

All cell culture reagents were purchased from Invitrogen (Darmstadt, Germany). PromoFectin® was from PromoCell (Heidelberg, Germany). Anti-pERK-1/2 (E-4), anti-EGR-1 (C-19) and anti-ERK-2 antiserum (C-14) were obtained from Santa Cruz Biotechnology (Heidelberg, Germany). Monoclonal anti-mouse/rat-CCR2 antibody, conjugated to allophycocyanin (APC) and the CCL2 ELISA kit were obtained from R&D systems (Wiesbaden, Germany) and peroxidase-conjugated anti-mouse or anti-rabbit antibody, both raised in goat, were obtained from Sigma-Aldrich (Deisenhofen, Germany). BSA, forskolin (FSK), poly-L-ornithine and poly-L-lysine were from Sigma-Aldrich and fura2-acetoxymethyl ester was obtained from Fluka (Deisenhofen, Germany). Coelenterazine H was purchased from Biaffin (Kassel, Germany) and firefly luciferase substrate was from Promega (Mannheim, Germany). [^3^H]-adenine was obtained from PerkinElmer (Rodgau, Germany). BAM8-22 and bradykinin (BK) were purchased from Biotrend (Cologne, Germany) and rat CCL2 from Peprotech (Hamburg, Germany). Cyclosporine A (CsA) was purchased from Sigma-Aldrich and PD-184352 was from Enzo life science (Lörrach, Germany). The generation of expression vectors encoding the human MRGPR-X1 (accession number: AF474990), the rat MRGPR-C (accession number: AF518245) or the murine MRGPR-C (accession number: AY152435) fused at the N-terminus to the Xpress epitope (pcDNA4-MRGPR-X1, pcDNA4-rMRGPR-C and pcDNA4-mMRGPR-C) was previously described [6]. The plasmid pG5α [24] encoding aequorin was kindly provided by Dr. Vladimir Chubanov (Walther-Straub-Institute, Munich, Germany). The reporter gene vector p5xSRE, carrying five repeats of the TCF/SRF-activated SRE-site (5′-GGATGTCCATATTAGGAC-ATC-3′) was kindly provided by Dr. Susanne Muehlich (Walther-Straub-Institute, Munich, Germany). The reporter gene vector p6xCRE carrying six repeats of the CREB-activated CRE site and the reporter gene vector pGL4.30 carrying four repeats of the NFAT-activated transcription site were obtained from Promega.

### Cell culture and transfections

HEK293 cells were cultured in DMEM (10% FBS, 2 mM L-glutamine, penicillin/streptomycin) and F11 (rat DRG neurons × murine NG18TG-2) cells in HAM's F-12 (15% FBS, 2 mM L-glutamine, hypoxanthine-aminopterin-thymidine, G-418 and penicillin/streptomycin). F11 cells were generated by Francel et al., [25] and kindly provided by Dr. Mederos y Schnitzler (Philipps University, Marburg, Germany). The CTMC line LAD-2 [26] was kindly provided from Dr. Arnold S. Kirshenbaum (National Institute of Allergy and Infectious Diseases, Bethesda, USA) and cultured in StemPro 34 (2 mM L-glutamine, nutrient supplement, 100 ng/ml recombinant human stem cell factor, penicillin/streptomycin). HEK293-MRGPR-X1, HEK293-rMRGPR-C, HEK293-mMRGPR-C and F11-MRGPR-X1 cells were described previously [6]. HEK293 cells stably expressing either receptor were subjected to a second selection (250 µg/ml G418 and 50 µg/ml zeocin) after transfection with the calcium sensing aequorin-eGFP construct pG5α to give HEK293-MRGPR-X1-Ae, HEK293-rMRGPR-C-Ae and HEK293-mMRGPR-C-Ae cells, respectively. The expression of the fusion proteins was controlled by measuring agonist-induced calcium releases and by fluorescence microscopy. Primary DRG neurons from day 18 embryonic Sprague/Dawley rats were purchased from Innoprot (Derio – Bizkaia, Spain) and cultured in NeuroBasal medium (B-27 supplement, 10% FBS, 2 mM L-glutamine, penicillin/streptomycin/neomycin) on poly-L-ornithine-coated well plates. For transient protein expression in HEK293 cells or DRG neurons PromoFectin® reagent was used according to the manufacturer's protocol. F11 cells were transfected using the Neon® transfection system from Invitrogen accordingly to settings provided by the manufacturer.

### Fura2-based single cell calcium imaging

24 h prior to the experiment F11-MRGPR-X1 cells were seeded at a density of 4–6×10^4^ cells on glass cover slips in 6-well plates coated with 0.1% poly-L-lysine. Cells were loaded with 5 µM fura2-AM in HEPES-buffered saline (HBS, 10 mM HEPES, 5 mM KCl, 1 mM MgCl_2_, 140 mM NaCl, 0.1% glucose and 2 mM CaCl_2_ adjusted to pH 7.4 with 1 M NaOH) supplemented with 0.02% pluronic F-127. Cover slips were washed twice with HBS without BSA and placed in a recording chamber. Cells were analyzed using a Polychrome 5000 monochromator (Till-Photonics, Gräfelfing, Germany) and an Andor charge-coupled device camera coupled to an inverted microscope (IX71, Olympus, Hamburg, Germany) by alternately exciting with 340 nm or 380 nm, respectively, every 0.5 s. Normalized fura2-ratios (340/380) were then plotted against the time in seconds.

### Fura2-based calcium measurements with a plate reader

Calcium transients in MRGPR-X1 or rodent MRGPR-C expressing HEK293, F11-MRGPR-X1 or LAD-2 cells were measured with a FLUOstar® Omega plate reader (BMG Labtech, Ortenberg, Germany) as described previously [6] using the fluorescent calcium indicator fura2. To determine concentration-response curves, integrated calcium signals were calculated, normalized to responses to maximal ligand concentrations (100%) and then subjected to curve fitting.

### Aequorin-based calcium measurements

Total luminescence in HEK293-MRGPR-Ae cells or DRG neurons transfected with the aequorin encoding plasmid G5α was measured using a FLUOstar® Omega plate reader at 37°C after labelling of the cells with coelenterazine H (5 µM) for 30 min at room temperature. HBS as a control or HBS including BAM8-22 was automatically injected 5 s after starting the measurement. In intervals of 1 s total emission was measured and is given as arbitrary units against the time in seconds.

### Western-Blotting

Cells were serum-starved for 24 h, stimulated for the indicated period of time with BAM8-22 or BK and then lysed in laemmli buffer. After SDS-PAGE and protein transfer to nitrocellulose, ERK-1/2 phosphorylation or EGR-1 expression was analyzed by using either a phospho-specific ERK-1/2 (p-ERK-1/2: 1∶3,000) or an EGR-1 specific (EGR1: 1∶10,000) antiserum and the corresponding peroxidase-coupled secondary antibody (1∶10,000). Membranes were stripped and then re-probed with an anti-ERK-2 antibody (t-ERK-2: 1∶10,000) and the corresponding peroxidase-coupled secondary antibody (1∶10,000). Immuno-reactivity was quantified by densitometry, ratios between p-ERK-1/2 or EGR-1 and t-ERK-2 signals were calculated, and ligand-induced ERK-1/2 phosphorylation/EGR-1 expression was normalized to not stimulated cells.

### Reporter gene assays

After transfection of a firefly luciferase reporter gene construct DRG neurons were serum-starved for 1 h and F11 or HEK293 cells for 24 h. Afterwards cells were stimulated with BAM8-22, BK or FSK for the indicated time in serum-free medium. Small molecule inhibitors (concentration as indicated) and the respective carrier control were pre-incubated for 30 minutes before addition of agonist and present during ligand stimulation. After stimulation cells were lysed (25 mM Tris/HCl pH 7.4, 4 mM EGTA, 8 mM MgCl_2_, 1 mM DTT and 1% Triton-X-100) and luciferase activity measured in white bottom 96-well plates after automatically injecting luciferase substrate and simultaneous detection of total light emission for 7.5 s post injection in a FLUOstar® Omega plate reader. Maximal light emission during this period was recorded and is indicated relative to not stimulated cells.

### RT-PCR

DRG neurons were serum-starved for 1 h and F11 or HEK293 cells for 24 h. Afterwards cells were stimulated with BAM8-22 or BK for the indicated period of time in serum-free medium. Total RNA from these cells or from not stimulated LAD-2 cells was isolated using the Tri reagent® (Sigma-Aldrich) according to the manufacturer's instructions. First strand synthesis was carried out with oligo(dT)_18_ primer using 2 µg of total RNA and the RevertAid^™^ H Minus First Strand cDNA Synthesis Kit (Fermentas, Sankt-Leon Roth, Germany). Quantitative RT-PCR (RTQ-PCR) was done using the LightCycler® 480 SybrGreen I Master Mix (Roche, Mannheim, Germany), 0.08 µl of the first strand synthesis reaction and 1 µM of the primer pairs shown in table 1. Samples were analyzed in a LightCycler® 480 (Roche) using the following conditions: initial denaturation for 15 min at 94°C, 55 cycles of 94°C for 10 sec, 55°C for 10 sec and 72°C for 10 sec. Crossing points (Cp) were determined by the software supplied with the LightCycler® 480 and ligand-induced modulation of target gene expression was calculated relative to β-actin using the ΔΔCp method. Non-quantitative RT-PCR was conducted in cDNA from LAD-2 cells using specific primers for MRGPR-X1 (forward: 5′-tgagtctctgatctgccctct-3′, reverse: 5′-tcaccagctgtatgatctctgatt-3′) or β-actin (forward: 5′-ccaaccgcgagaagatga-3′, reverse: 5′-ccagaggcgtacagggatag-3′). PCR settings were the same as in RTQ-PCR and the number of amplification cycles is indicated in the figure legend.

**Table 1:** Primer sequences used for RTQ-PCR experiments.

gene number	name	forward primer	reverse primer
NM_031144.2	β-actin	Ggctcctagcaccatgaaga	atctgctggaaggtggacag
NM_022197.2	c-Fos	Gggacagcctttcctactacc	gatctgcgcaaaagtcctgt
NM_001013146.1	FosB	Acgccgagtcctactccag	tctcctcctcttcgggagac
NM_001109302.1	SRF	Gcacagacctcacgcaga	atgtggccacccacagtt
NM_021836.2	JunB	Gggactgggagctcatacc	aaagggtggtgcatgtgg
NM_012551.2	EGR-1	Cgaacaaccctacgagcac	gcgccttctcgttattcaga
NM_053633.1	EGR-2	Ctacccggtggaagacctc	aatgttgatcatgccatctcc
NM_017086.1	EGR-3	Caatctgtaccccgaggaga	ccgatgtccatcacattctct
NM_019137.1	EGR-4	Gccctcttcaacctcatgtct	ggggaaagggacatccag
NM_024388.1	Nur77	Tgttgatgttcctgcctttg	ggaggccatgtcgatcag
NM_001106009.1	Ldb2	Cagtgctgggaacacaacc	ttggctctcctaccaccatc
NM_017334.1	Crem2	Ctgctttgccacaaggtgt	cgacattctttagcagcttcc
NM_001110860.1	Crem3	Actagcacggggcaatacat	accatcagatcctgggttagaa
NM_153626.1	Npas4	Agggtttgctgatgagttgc	tcccctccacttccatctt
NM_031327.2	Cyr61	Ggatctgtgaagtgcgtcct	ctgcatttcttgcccttttt
NM_022266.2	Ctgf	gctgacctagaggaaaacattaaga	ccggtaggtcttcacactgg
NM_021866.1	CCR2	aagaagtatccaagagcttgatgag	tcaccatcatcatagtcatacgg
NM_001007235.1	IP3R-1	Catccagtatggcaacgtga	tcatggcgttcttctccagt
NM_031530.1	CCL2	Agcatccacgtgctgtctc	gatcatcttgccagtgaatgag
NM_017232.3	COX-2	tccaacctctcctactacaccag	tccagaacttcttttgaatcagg
NM_012513.3	BDNF	Agcgcgaatgtgttagtggt	gcaattgtttgcctctttttct

### Flow cytometry

Cells were serum-starved for 4 h and stimulated for 20 h in serum-free medium with BAM8-22, detached with PBS/2 mM EDTA, washed once with PBS/10% FBS and stained with anti-rat-CCR2-APC antibody or the corresponding isotype for 30 minutes at room temperature. After two washing steps cells were resuspended in PBS/10% FBS and analyzed by flow cytometry (CyAn ADP, Beckman Coulter).

### cAMP accumulation assay

To determine CCL2-promoted inhibition of forskolin-induced cAMP accumulation, F11 cells were seeded in 12-plates dishes coated with 0.1% polyl-L-lysine 24 h prior to the experiment. Afterwards cells were serum-starved for 4 h, stimulated for 20 h with BAM8-22 or BK in serum-free HAM's F-12 medium containing 2 µCi/ml of [^3^H] adenine. Cells were stimulated for 30 min at 37°C in HAM's F-12 containing 100 mM HEPES, pH 7.4, 2.5 µM IBMX, 5 µM forskolin alone or along with 100 ng/ml CCL2. The reaction was terminated by removing the medium and adding ice-cold 5% trichloroacetic acid to the cells. [^3^H] cAMP was then purified by sequential chromatography over dowex resin and aluminium oxide, and the cAMP accumulation expressed as the total amount of [^3^H] cAMP in DPM/well.

### Data analysis

Data were analyzed using Prism4.0 (GraphPad Software Inc., San Diego, CA). Statistical significance of differences was assessed by the two-tailed Student's t-test (two groups) or by one-way analysis of variance and Tukey's honest significance post-hoc test (more than two groups). Asterisks (***p<0.001, **p<0.01, *p<0.05) or dagger signs (^†††^p<0.001, ^††^p<0.01, ^†^p<0.05) were used to indicate significant differences between different experimental groups.

## Results

### BAM8-22-induced calcium release and ERK-1/2 activation in HEK293-MRGPR-X1 and F11-MRGPR-X1 cells

**Figure 1:**
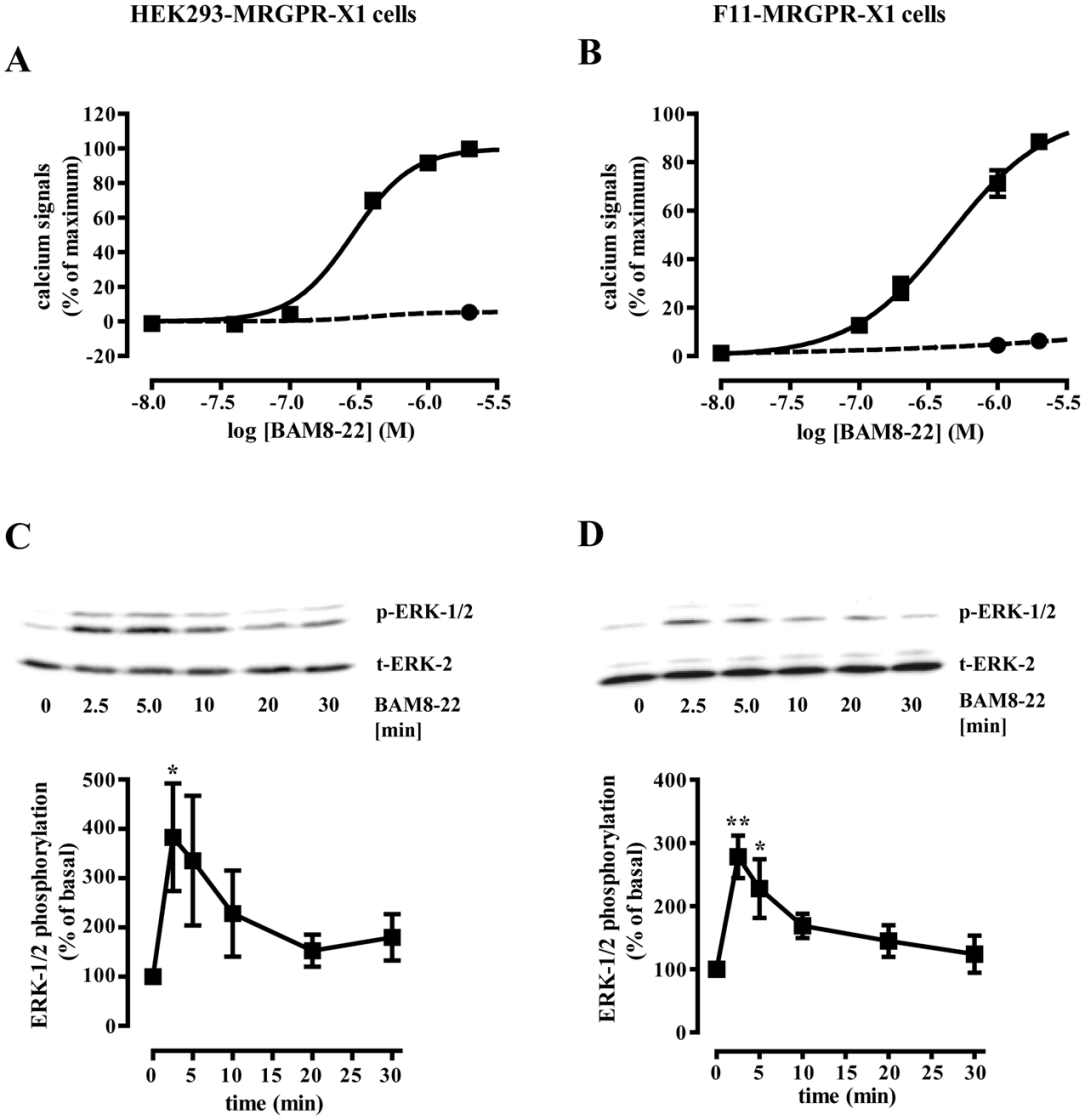
MRGPR-X1 stimulate ERK-1/2 in HEK293- and F11-MRGPR-X1 cells. Concentration response curves of BAM8-22-promoted calcium signals in fura2-loaded HEK293-MRGPR-X1 (A) or in F11-MRGPR-X1 (B) cells (squares) or mock cells (circles) are shown. BAM8-22-induced (1 µM) phosphorylation of ERK-1/2 in HEK293-MRGPR-X1 (C) or in F11-MRGPR-X1 (D) cells was analyzed by western-blotting using a phospho-specific antibody (p-ERK-1/2). Afterwards blots were stripped and re-probed with an antibody against ERK-2 (t-ERK-2). One representative blot is shown. Ligand-induced ERK-1/2 phosphorylation was quantified by densitometry and is given normalized to not stimulated cells. Data from 4 experiments were compiled and expressed as the mean ± S.E.M. Asterisks indicate significant differences to not stimulated cells.

Binding of the specific agonist BAM8-22 to MRGPR-X1 has been described to activate G_q_ proteins and thus, to increase intracellular calcium concentrations [1], [6], [7]. Therefore, we first analyzed BAM8-22-induced calcium signals in previously reported HEK293-MRGPR-X1 or F11-MRGPR-X1 cells that stably express low levels of MRGPR-X1 [6], [8]. Stimulation of MRGPR-X1 with BAM8-22 elicited calcium transients with a potency of ∼200 nM (Fig. 1A+B) consistent with previous reports [1], [5]. ERK-1/2 play a central role in GPCR-promoted gene induction, because ERK-1/2 enhance CREB- and TCF/SRF-dependent gene expression. Thus, we also analyzed BAM8-22-promoted ERK-1/2 phosphorylation in HEK293-MRGPR-X1 and F11-MRGPR-X1 cells. We observed marked, but transient phosphorylation of ERK-1/2 in both cell pools, with a peak at 5 min and a decline thereafter (Fig. 1C+D). Given that calcium has been reported to enhance NFAT-dependent gene expression via calcineurin, and ERK-1/2 via TCF/SRF or CREB, we postulated that BAM8-22 might induce CREB-, TCF/SRF- or NFAT-dependent gene expression in MRGPR-X1 expressing cells.

### BAM8-22-induced activation of TCF/SRF- and NFAT-dependent reporters in HEK293-MRGPR-X1 and F11-MRGPR-X1 cells

**Figure 2:**
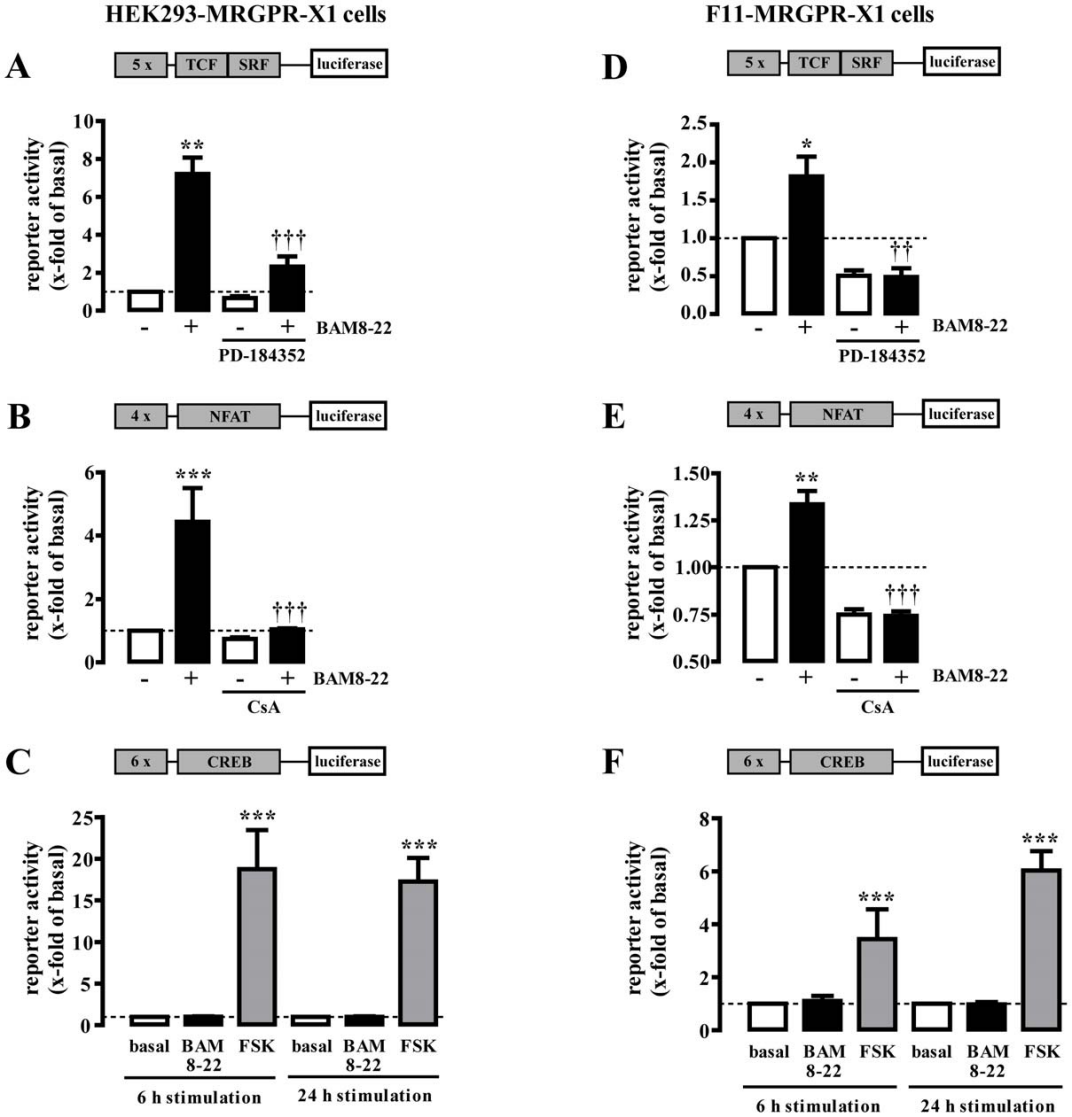
MRGPR-X1 stimulate TCF/SRF- and NFAT-dependent reporter in HEK293- and F11-MRGPR-X1 cells. TCF/SRF-, NFAT- or CREB-dependent reporter gene constructs were transfected into HEK293-MRGPR-X1 (A–C) or in F11-MRGPR-X1 (D–F) cells. Cells were stimulated with 1 µM BAM8-22 for 6 h or as indicated. 5 µM FSK was used as a control for the CREB reporter. CsA (1 µM, 30 min) was used to block the NFAT activator calcineurin and PD-184352 (10 µM, 30 min) to block ERK-1/2 activity. Data from 3–5 independent experiments performed in triplicates were compiled and expressed as the mean ± S.E.M. Asterisks indicate significant differences to not stimulated cells. Dagger signs indicate significant differences between BAM8-22-stimulated inhibitor-treated and untreated cells.

To test the ability of BAM8-22 to induce gene expression via MRGPR-X1, we used HEK293-MRGPR-X1 and F11-MRGPR-X1 cells to perform reporter gene assays employing constructs that contained binding sites for TCF/SRF, NFAT or CREB. Stimulation of both cell types with BAM8-22 led to activation of the TCF/SRF reporter (Fig. 2A+D) preventable by a specific ERK-1/2 inhibitor (PD-184352), and of the NFAT reporter sensitive to CsA, an inhibitor of the NFAT activator calcineurin (Fig. 2B+E). In contrast, BAM8-22-induced activation of the CREB reporter was not observed in either MRGPR-X1 expressing cell type (Fig. 2C+F).

### BAM8-22-induced expression of c-Fos and EGR-1 via ERK-1/2 in F11-MRGPR-X1 cells

**Figure 3:**
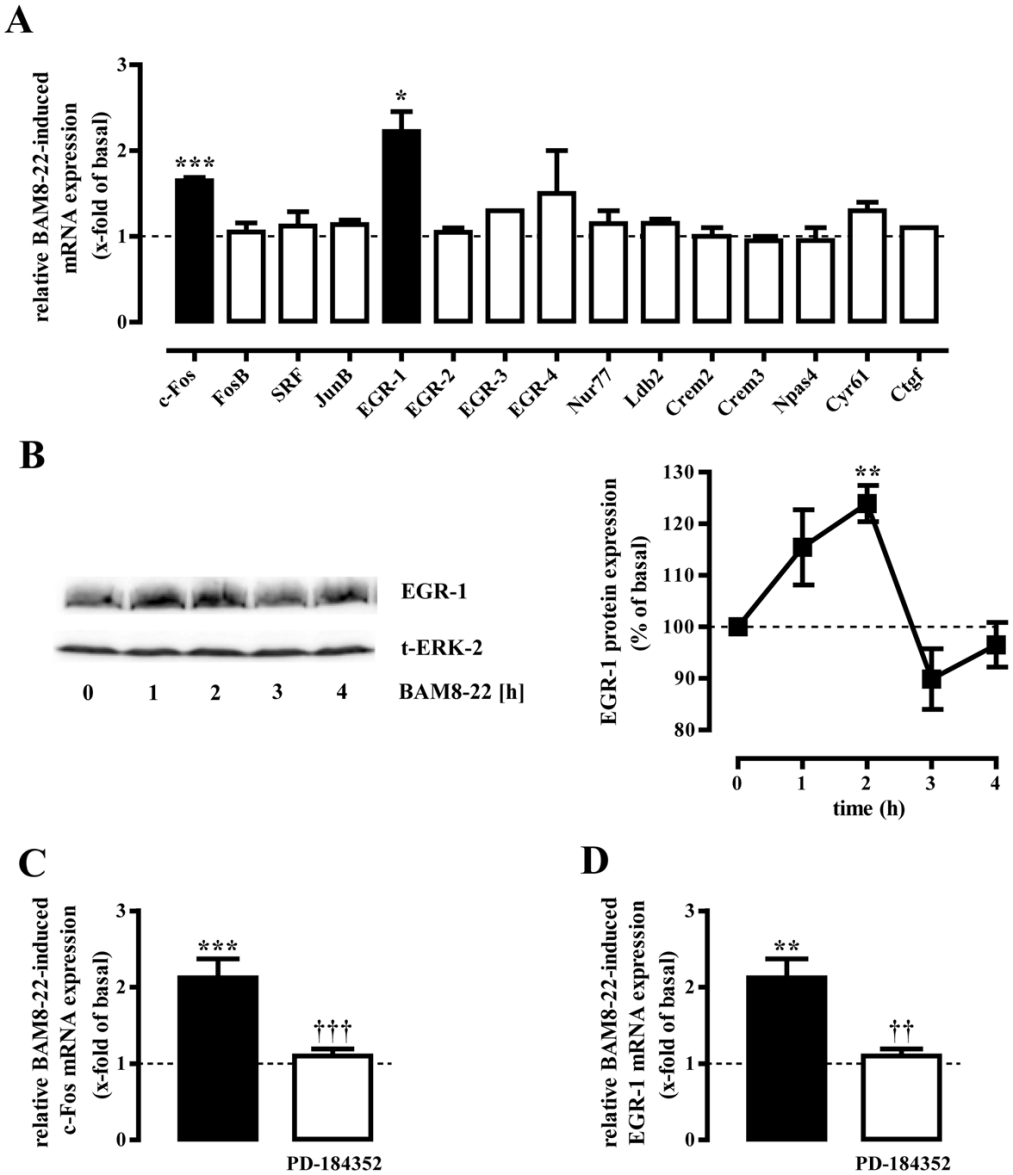
MRGPR-X1 induce EGR-1 via ERK-1/2 in F11-MRGPR-X1 cells. RTQ-PCR experiments were performed with cDNAs derived from serum-starved F11-MRGPR-X1 cells stimulated or not with BAM8-22 (1 µM) for 1 h (A, C and D) using specific primers for 15 distinct genes as indicated in table 1. The name of the analyzed gene is listed beneath the corresponding bar. Genes with a p value <0.05 are show in black bars. In (C and D) cells were treated or not with PD-184352 (30 min, 10 µM). Relative BAM8-22-induced gene expression was normalized to β-actin and calculated using the ΔΔCp method. In (B) serum-starved F11-MRGPR-X1 cells were stimulated or not with BAM8-22 (1 µM) for the indicated period of time and expression of EGR-1 was determined by western-blotting. Afterwards blots were stripped and re-probed with an antibody against ERK-2 (t-ERK-2). One representative blot is shown. Ligand-induced EGR-1 expression was quantified by densitometry and is given normalized to not stimulated cells. Data from 4 independent experiments were compiled and expressed as the mean ± S.E.M. Asterisk indicate a significant difference to not stimulated cells. Dagger signs indicate a significant difference between BAM8-22-stimulated inhibitor-treated and untreated cells.

To gain deeper insight into BAM8-22-induced expression of TCF/SRF-dependent genes, we analyzed mRNA levels of 15 genes reported to be up-regulated by TCF/SRF in neurons [18]. As shown in figure 3A, mRNA levels of c-Fos and EGR-1 were significantly elevated in F11-MRGPR-X1 cells after 1 h of BAM8-22 stimulation and total EGR-1 protein levels significantly increased after challenging the cells with BAM8-22 for 1 to 2 h (Fig. 3B). Because c-Fos and EGR-1 mRNA induction was blocked by PD-184352 (Fig. 3C and D), we concluded that ERK-1/2 activity is required for BAM8-22-induced expression of TCF/SRF-dependent genes.

### BAM8-22-induced expression of IP3R-1 and CCR2 via calcineurin in F11-MRGPR-X1 cells

**Figure 4:**
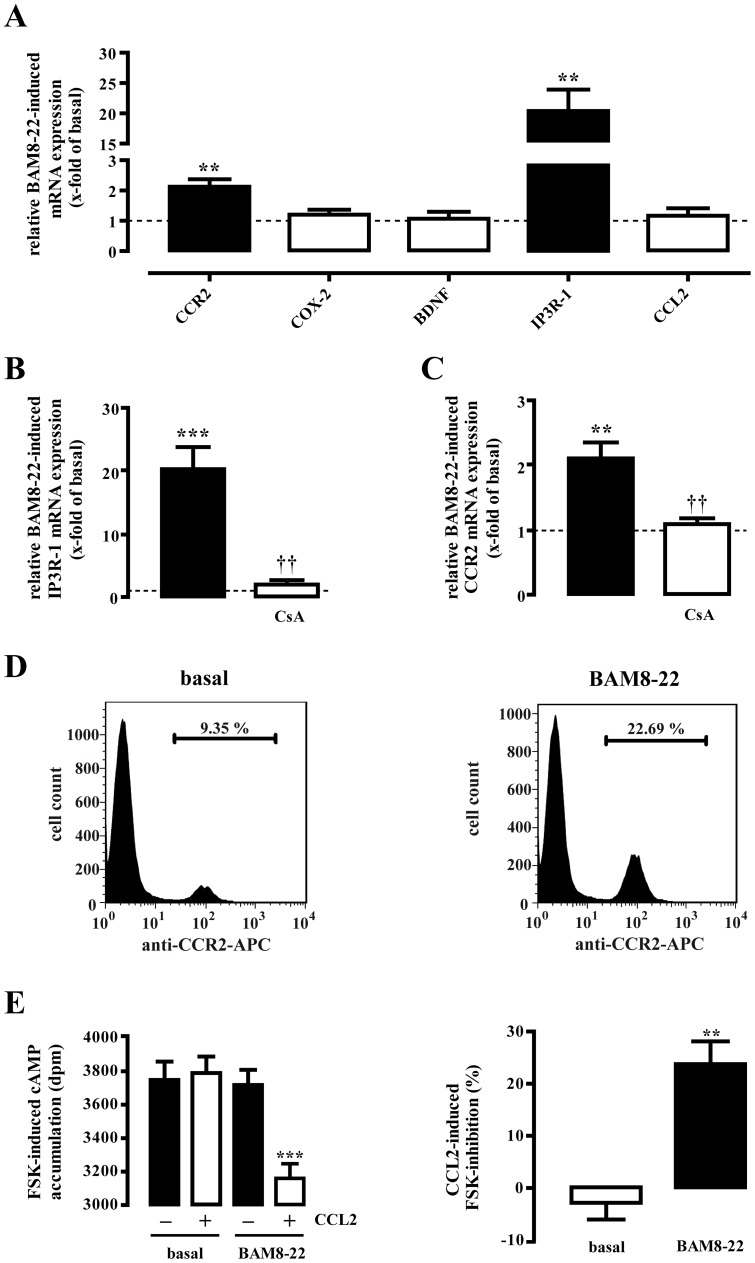
MRGPR-X1 induce CCR2 via NFAT in F11-MRGPR-X1 cells. RTQ-PCR experiments were performed with cDNAs derived from serum-starved F11-MRGPR-X1 cells stimulated or not with BAM8-22 (1 µM) for 6 h (A-C) using specific primers for 5 distinct genes as indicated in table 1. The name of the analyzed gene is listed beneath the corresponding bar. Genes with a p value <0.05 are show in black bars. In (B and C) cells were treated or not with CsA (1 µM, 30 min). Relative BAM8-22-induced gene expression was normalized to β-actin and calculated using the ΔΔCp method. Data from 4 independent experiments were compiled and expressed as the mean ± S.E.M. BAM8-22-induced (1 µM, 20 h) CCR2 protein expression in F11-MRGPR-X1 cells was assessed by flow cytometry (D) or by CCL2-promoted (100 ng/ml) inhibition of FSK-induced (5 µM) cAMP accumulation (E). In (D) one representative experiment is shown. Accumulation of the data from 5 independent experiments revealed an increase in the number of CCR2 positive cells by 10.8±1.4% after BAM8-22 stimulation. In (E, left panel) one representative experiment is shown and in (E, right panel) data from 5 independent experiments performed in triplicates were compiled and expressed as the mean ± S.E.M. Asterisks indicate a significant difference to not stimulated cells. Dagger signs indicate a significant difference between BAM8-22-stimulated inhibitor-treated and untreated cells.

Recently, it has been reported that CCR2, IP3R-1, CCL2, cyclooxygenase-2 (COX-2) or the brain-derived neurotrophic factor are up-regulated by NFAT in neurons [16], [17], [27]. Thus, we analyzed effects of BAM8-22 on the mRNA levels of these genes in F11-MRGPR-X1 cells. As shown in figure 4, IP3R-1 and CCR2 were up-regulated after 6 h of BAM8-22 incubation in a CsA-dependent manner, indicating that BAM8-22 induces expression of both genes via calcium/calcineurin-mediated NFAT activation in F11-MRGPR-X1 cells. BAM8-22-induced expression of CCR2 is of particular interest, because enhanced CCR2 expression in DRG neurons is strongly associated with various neuropathic pain syndromes [28]. To further corroborate our observation of BAM8-22-induced CCR2 mRNA expression, we performed flow cytometry experiments with F11-MRGPR-X1 cells and an APC-conjugated CCR2 specific antibody (Fig. 4D). Stimulation of cells with BAM8-22 increased the amount of CCR2 positive cells by 10.8±1.4%, demonstrating that BAM8-22-promoted induction of CCR2 mRNA is translated into increased expression of the CCR2 protein. To test whether BAM8-22-induced CCR2 expression was sufficient to affect the sensitivity of F11-MRGPR-X1 cells to CCL2, we took advantage of the G_i/o_ coupling of CCR2 and performed cAMP accumulation assays with BAM8-22-treated cells. As shown in figure 4E, untreated F11-MRGPR-X1 cells did not respond to CCL2, whereas BAM8-22-pre-treated F11-MRGPR-X1 cells significantly responded to the peptide, such that the CCR2 specific agonist reduced FSK-induced cAMP accumulation by 23.8±4.3%, comparable to the effects of various opioids acting on endogenous opioid receptors constitutively expressed in F11 cells [29]. Therefore, we concluded that BAM8-22 enhances the expression of functional CCR2 in F11-MRGPR-X1 cells, thus enabling CCL2 to engage cognate signaling pathways in DRG neuron-derived cells.

### Bradykinin induces EGR-1 expression via ERK-1/2 but not CCR2 expression via NFAT

**Figure 5:**
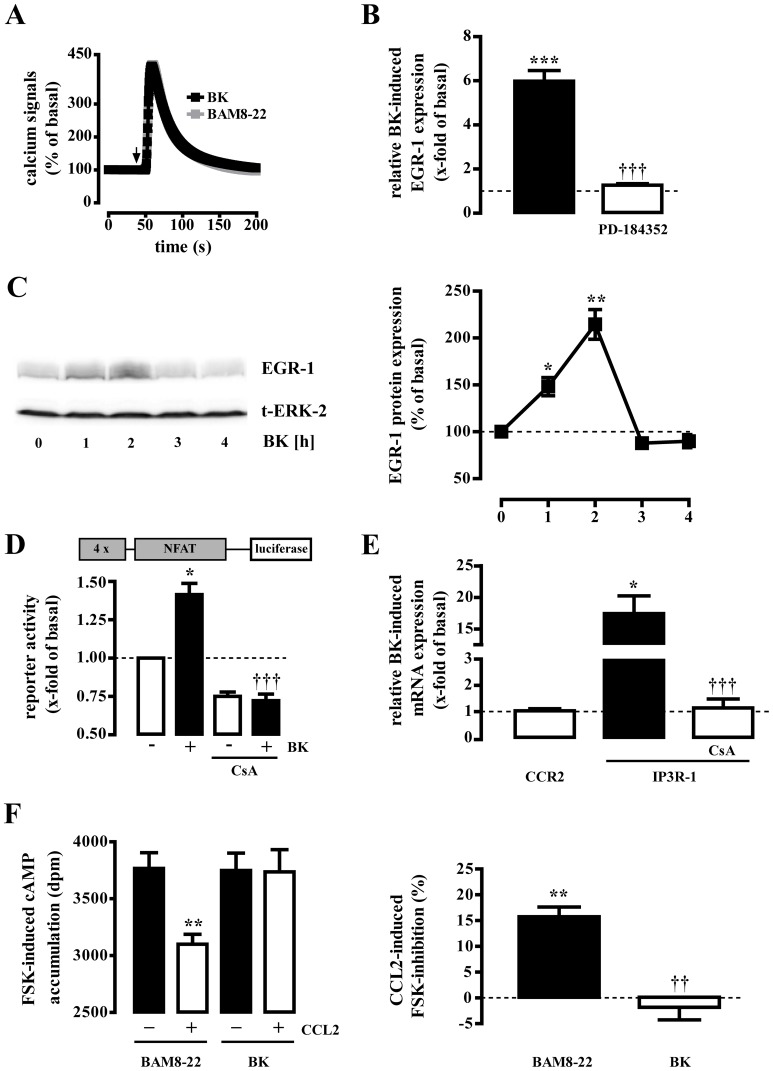
B2R do not induce CCR2 despite NFAT activation. (A) BAM8-22- (2 µM) or BK-induced (1 µM) calcium signals were determined in single fura2-loaded F11-MRGPR-X1 cells by calcium imaging. Data of ∼300 cells were compiled and expressed as the mean ± S.E.M. RTQ-PCR experiments were performed with cDNAs derived from serum-starved F11-MRGPR-X1 cells stimulated or not with BK (1 µM) for 1 h in (B) or 6 h in (E). Relative BK-induced gene expression was normalized to β-actin, calculated using the ΔΔCp method, and expressed as the mean ± S.E.M. (C) Serum-starved F11-MRGPR-X1 cells were stimulated or not with BK (1 µM) for the indicated period of time and expression of EGR-1 was determined by western-blotting. Afterwards blots were stripped and re-probed with an antibody against ERK-2 (t-ERK-2). One representative blot is shown. Ligand-induced EGR-1 expression was quantified by densitometry and is given normalized to not stimulated cells as the mean ± S.E.M. (D) BK-induced (1 µM, 6 h) activation of the NFAT reporter is shown in F11-MRGPR-X1 cells. Data are expressed as the mean ± S.E.M. PD-184352 (10 µM, 30 min) was used to inhibit ERK-1/2 activity in (B) or CsA (1 µM, 30 min) to block calcineurin in (D and E). In (B–E) 4 independent experiments were conducted, respectively. In (F) CCR2 protein expression in F11-MRGPR-X1 cells was assessed by CCL2-promoted (100 ng/ml) inhibition of FSK-induced (5 µM) cAMP accumulation after pre-stimulation of the cells with BAM8-22 or BK (1 µM, 20 h). In (F, left panel) one representative experiment is shown and in (F, right panel) data from 5 independent experiments performed in triplicates were compiled and expressed as the mean ± S.E.M. Asterisks indicate a significant difference to not stimulated cells. Dagger signs indicate a significant difference between BK-stimulated inhibitor-treated and untreated cells or between BK- and BAM8-22-stimulated cells.

Next we asked whether other G_q_ protein activating neuropeptides would also induce EGR-1 and CCR2 expression in a DRG neuron-derived cell model. F11 cells have been reported to endogenously express G_q_ activating bradykinin-2-receptors (B2R) [25]. Thus, we first compared calcium transients triggered by BAM8-22 or BK. As shown in figure 5A, calcium signals in F11-MRGPR-X1 cells induced by saturated concentrations (EC_95_) of BK or BAM8-22 were almost identical illustrating that activation of recombinant MRGPR-X1 did not elicit unphysiological high calcium transients. Furthermore, BK induced EGR-1 expression (Fig. 5B+C) in accord with previously observed effects of BK on EGR-1 expression in human fibroblasts or NG108-15 neuroblastoma cells [30], [31]. BK also activated the NFAT reporter (Fig. 5D) and also induced IP3R-1 mRNA expression via NFAT (Fig. 5E). However, despite these similarities, BK treatment did neither induce CCR2 mRNA expression (Fig. 5E), nor did it impart CCL2 sensitivity to F11-MRGPR-X1 cells (Fig. 5F), suggesting that CCR2 induction is not a common feature among G_q_ activating neuropeptides, but a characteristic property of MRGPR-X1.

### BAM8-22-induced CCR2 and EGR-1 mRNA expression in cultured rat DRG neurons

**Figure 6:**
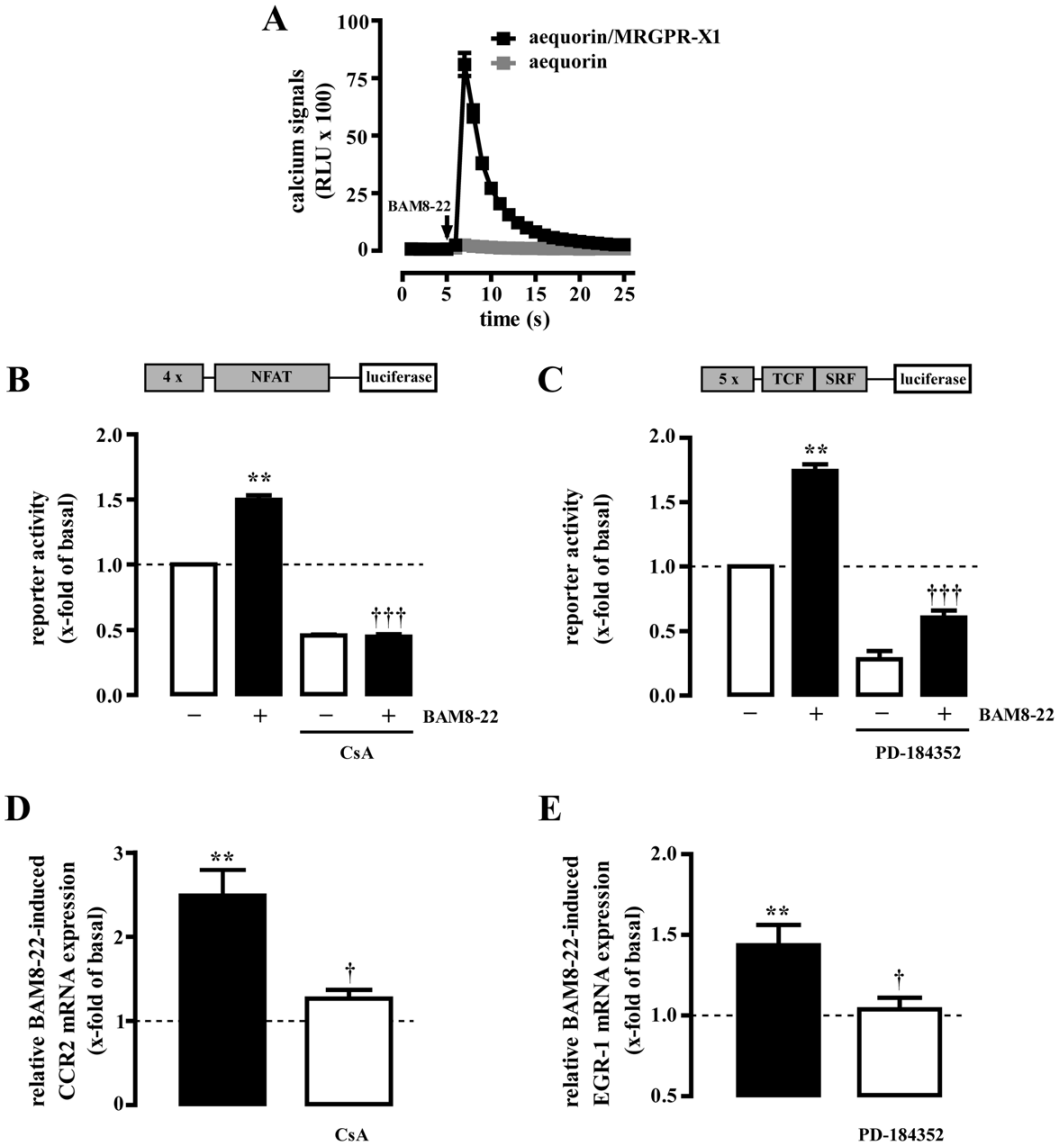
MRGPR-X1 induce EGR-1 via ERK-1/2 and CCR2 via NFAT in primary DRG neurons. BAM8-22-induced (2 µM) calcium signals in rat DRG neurons co-expressing MRGPR-X1 and aequorin or solely aequorin are presented in (A). BAM8-22-induced (2 µM, 8 h) activation of the NFAT (B) or TCF/SRF (C) reporter is shown in rat DRG neurons transiently co-expressing MRGPR-X1. RTQ-PCR experiments were performed with cDNAs derived from serum-starved MRGPR-X1 expressing rat DRG neurons stimulated or not with BAM8-22 (2 µM) for 6 h (D) or 40 min (E). CsA (1 µM, 30 min) was used to block calcineurin in (B and D) or PD-184352 (10 µM, 30 min) to inhibit ERK-1/2 activity in (C and E). Relative BAM8-22-induced gene expression was normalized to β-actin and calculated using the ΔΔCp method. Data from 4 independent experiments were compiled and expressed as the mean ± S.E.M. Asterisks indicate a significant difference to not stimulated cells. Dagger signs indicate a significant difference between BAM8-22-stimulated inhibitor-treated and untreated cells.

To substantiate our observations that MRGPR-X1 induce expression of chronic pain markers, we used DRG neurons from day 18 embryonic rats and transfected them with MRGPR-X1 cDNA after 10–14 days in culture. As shown in figure 6A, only MRGPR-X1 expressing DRG neurons reacted to BAM8-22 with robust calcium signals. Furthermore, similar to F11-MRGPR-X1 cells, stimulation of MRGPR-X1 expressing DRG neurons with BAM8-22 increased the activity of the NFAT (Fig. 6B) and the TCF/SRF reporter (Fig. 6C), suggesting that enhancing effects of BAM8-22 on gene expression are not restricted to the F11 cell line, but also occur in cultured DRG neurons. In line with this notion, BAM8-22 also elevated CCR2 mRNA levels in a CsA-sensitive (Fig. 6D) and EGR-1 transcripts in a PD-184352-dependent manner (Fig. 6E), supporting our finding that BAM8-22 induces CCR2 and EGR-1 expression in DRG neurons.

### BAM8-22-promoted CCL2 release in LAD-2 cells

**Figure 7:**
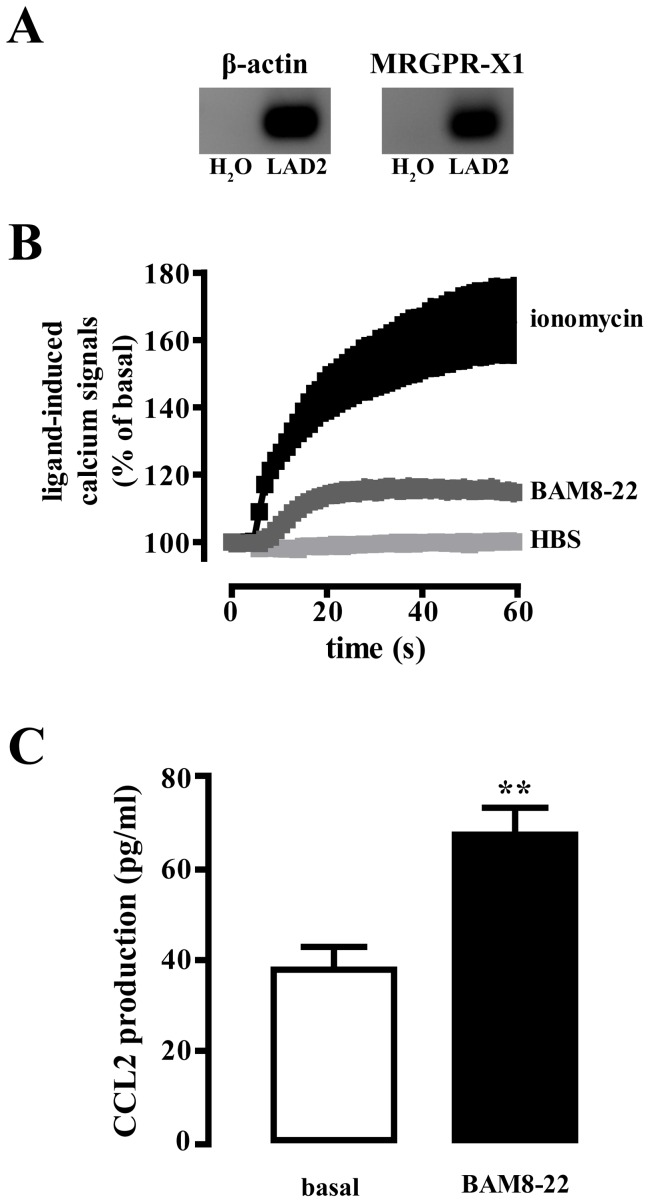
MRGPR-X1-induced CCL2 release in LAD-2 mast cells. (A) MRGPR-X1 (40 cycles) or β-actin (30 cycles) mRNA expression was determined in LAD-2 cells by RT-PCR. As a negative control, RT-PCR was conducted without addition of cDNA (H_2_O). (B) Calcium signals in fura2-loaded LAD-2 cells are shown after injection of BAM8-22 (5 µM) or ionomycin (5 µM) or HBS as positive or negative control, respectively. (C) CCL2 release in LAD-2 cells after stimulation with BAM8-22 (5 µM, 18 h) was determined by ELISA. Data from 4 independent experiments performed in triplicates were compiled and expressed as the mean ± S.E.M. Asterisks indicate a significant difference to not stimulated cells.

So far, MRGPR-X1 are thought to be exclusively expressed in DRG neurons and therefore were originally termed sensory neuron-specific GPCR [1]. Recently, significant mRNA levels of MRGPR-X1 were detected in native CTMC and in the human LAD-2 mast cell line [4]. In theses lines, we detected significant MRGPR-X1 transcripts in LAD-2-derived cDNA samples (Fig. 7A). However, activation of the MRGPR-X1 protein by its specific agonist BAM8-22 has not been demonstrated yet. Thus, we took advantage of LAD-2 cells and performed fura2-based calcium measurements in order to monitor activation of the MRGPR-X1 protein by its specific agonist BAM8-22. As shown in figure 7B, BAM8-22 induced robust calcium transients in LAD-2 cells, indicating endogenous expression of functional MRGPR-X1. Next, we asked whether BAM8-22-induced signaling would affect CCL2 release in LAD-2 cells. Notably, when performing ELISA assays with an antibody specific to human CCL2, we observed that the chemokine was released into the supernatant of LAD-2 cells in response to BAM8-22 (Fig. 7C), indicating that MRGPR-X1 exert their action in cells of the peripheral nervous and the immune system.

## Discussion

Primate-specific MRGPR-X1 are selectively expressed in DRG neurons and therefore represent promising targets for innovative pain therapies [1], [2]. Interestingly, disease-causing genes are strongly enriched among primate-specific genes [32] and the ligand-binding domain of MRGPR-X1 has been described as a product of strong positive selection [33], [34], further underscoring the potential of MRGPR-X1 as therapeutic targets. Despite the high therapeutic potential of MRGPR-X1, hardly any information about the role of this receptor subtype in pain chronification is available. Herein, we analyzed BAM8-22-induced gene expression in HEK293 and F11 cells stably expressing MRGPR-X1. We observed activation of a NFAT- and TCF/SRF-, but not of a CREB-dependent reporter. In-depth analysis of F11-MRGPR-X1 cells confirmed these findings, because BAM8-22-induced ERK-1/2 or calcineurin activation led to significant up-regulation of c-Fos, EGR-1, IP3R-1 and CCR2 mRNA. Thus, our data provide first evidence of BAM8-22-induced gene expression in DRG neuron-derived cells. F11 cells emerged as a valuable biological tool for the analysis of DRG neuron-related signaling pathways, since many studies reported striking similarities to cultured DRG neurons [25], [27], [35], [36], [37], [38], [39], [40], [41], [42], [43]. Along these lines, data from our laboratory revealed morphine-induced expression of the early growth response protein-4 via µ opioid receptors in F11 cells and cultured rat DRG neurons [29]. In the same vein, we confirmed BAM8-22-promoted activation of the NFAT or TCF/SRF reporter and enhanced CCR2 or EGR-1 mRNA expression in cultured rat DRG neurons transiently expressing MRGPR-X1.

### BAM8-22-induced EGR-1 expression via ERK-1/2

Activated TCF/SRF complexes have been shown to induce immediate early genes such as c-Fos, c-jun or members of the EGR family in neurons of the peripheral or central nervous system and thereby modulate synaptic activity, memory and learning [18]. Herein, we observed BAM8-22-promoted up-regulation of c-Fos and EGR-1 in F11-MRGPR-X1 cells. Induction of c-Fos in the spinal dorsal horn is an established marker of neuronal activity or noxious stimulation [44]. However, less is known about the role of c-Fos induction in DRG neurons. A recent study correlated surgery- or opioid-induced hyperalgesia with ERK-1/2 phosphorylation and c-Fos expression in DRG neurons [45], suggesting that BAM8-22-promoted c-Fos induction is in good agreement with enhanced pain sensations previously reported after BAM8-22 application in humans [9]. So far, induction of the zinc-finger transcription factor EGR-1 (also *zif/268*) in DRG neurons has been described for neurotrophins such as the nerve growth factor or tumor necrosis factor-α [46], [47]. Tumor necrosis factor-α-promoted EGR-1 expression plays a significant role in carrageenan-induced inflammatory hyperalgesia [46] and selective knockdown of the EGR-1 protein significantly reduced inflammatory pain induced by complete Freund's adjuvants [48]. Furthermore, elevated EGR-1 protein levels correlate with inflammatory pain in sheep [49], strongly indicating a major contribution of EGR-1 to inflammatory pain in various species.

### BAM8-22-induced CCR-2 expression via calcineurin

NFAT-induced gene expression significantly contributes to the differentiation and proliferation of lymphocytes. Recent work suggested that NFAT activation also increases the expression of pro-nociceptive genes such as COX-2 or CCR2 in neurons [17], [27], indicating a significant role of NFAT-dependent gene induction for pain chronification [28]. Induction of IP3R-1 via NFAT, as shown herein for G_q_-coupled MRGPR-X1, has so far been linked to the activation of receptor tyrosine kinases by neurotrophins in DRG, spinal cord and hippocampal pyramidal neurons [50] or to potassium-induced depolarisation in rat cerebellar neurons [51]. Thus, IP3R-1 is one of the best described targets of NFAT-dependent gene expression in non-immune cells, but cellular or physiological consequences of enhanced IP3R-1 expression levels in neurons have not been sorted out. Chemically induced calcium influx in F11 cells or DRG neurons by ionomycin or high potassium concentrations have been reported to induce CCR2 mRNA expression via NFAT [27]. However, G protein-promoted CCR2 induction via NFAT has not yet been described. Here, we report that stimulation of F11-MRGPR-X1 cells with BAM8-22 not only increased CCR2 mRNA and protein levels, but also imparted sensitivity to the endogenous CCR2 agonist, CCL2, to these cells. MRGPR-X1-induced CCR2 expression is of particular interest, considering that CCL2-induced activation of CCR2 in DRG neurons plays a major role in distinct neuropathic pain syndromes, as demonstrated by significantly diminished neuropathic pain responses found in mice deficient of the CCR2 gene [52] and by abolished neuropathy-induced hyperalgesia and allodynia after application of CCR2 specific antagonists to wild-type mice [22], [53]. Therefore, inhibition of CCL2-promoted CCR2 signaling is a promising approach towards a future therapy of neuropathic pain. However, because of versatile physiological roles of chemokines significant side effects are expected when blocking CCR2-induced signaling *in vivo*. Of note, CCR2-deficient mice exhibited normal pain behaviour under physiological conditions and many studies showed that, similar to F11-MRGPR-X1 cells, CCR2 expression levels in DRG neurons are rather low under non-neuropathic conditions [27], [28]. Hence, CCR2 up-regulation in DRG neurons emerged as a key event that initiates neuropathic pain syndromes or enhances their progress [22], [23], [54], [55], [56], [57]. Within this model, controlling CCR2 expression selectively in DRG neurons would be a promising approach to block chemokine-promoted signaling and thus, to alleviate neuropathic pain syndromes without affecting CCL2/CCR2 signaling in other cell types. Herein, we define MRGPR-X1 as endogenous inducers of CCR2 in DRG neurons. Thus, although our conclusions still have to await ultimate proof by *in vivo* studies in primates, we propose a role of MRGPR-X1 in chemokine-promoted pain chronification and consider MRGPR-X1 as promising targets to selectively manipulate CCR2 expression in DRG neurons.

### CCR-2 expression via calcineurin is not common among G_q_ activating neuropeptides

Interestingly, BK-promoted NFAT activation did not induce CCR2 expression in F11 cells, although IP3R-1 expression was found to be significantly induced as was COX-2 expression in DRG neurons [16]. *Vice versa*, BAM8-22-induced NFAT activation led to increased CCR2, but not to COX-2 expression (Fig. 4A). Thus, we postulate that NFAT up-regulates pro-nociceptive genes in DRG neurons in concert with other transcription factors and therefore is required, but not sufficient to mediate CCR2 expression via MRGPR-X1 or COX-2 induction via B2R. Further, in contrast to B2R, MRGPR-X1 are resistant against agonist-promoted internalisation and thus, do not interact with arrestins, which have been reported to affect GPCR-promoted gene induction [6], [58], [59]. Therefore, distinct effects of B2R and MRGPR-X1 on gene expression could either be due to the sustained effects of non-desensitizing MRGPR-X1-promoted signaling or caused by the functional interactions between B2R and arrestins. At last, it has to be mentioned that B2R were endogenously and MRGPR-X1 recombinantly expressed in F11 cells. Recombinant protein expression often leads to significant higher protein levels compared to endogenous expression. Unfortunately, absolute receptor numbers of MRGPR-X1 cannot be determined, because no radiolabeled BAM8-22 is commercially available. Thus, we cannot completely rule out that distinct protein expression levels are responsible for the differences observed. However, it is noteworthy that calcium signaling induced by both receptors and saturated agonist concentrations were almost identical, suggesting comparable numbers of both receptors. Hence, additional work is required to find out how distinct neuropeptides differentially induce NFAT-dependent genes.

### BAM8-22-induced CCL2 release in mast cells

**Figure 8:**
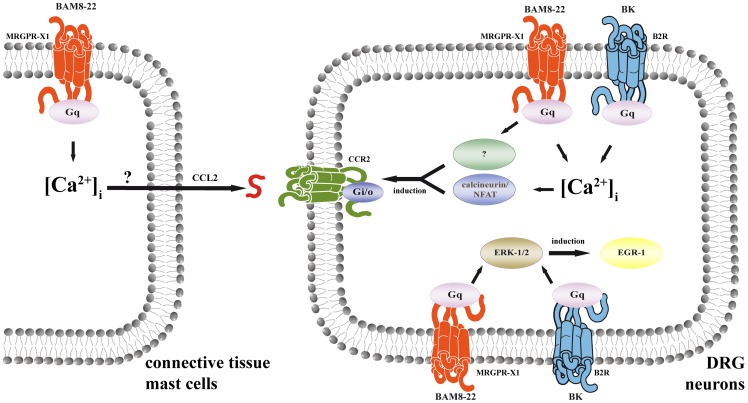
MRGPR-X1-induced signaling in DRG neurons and CTMCs. A cartoon illustrating the signaling circuit by which MRGPR-X1 affect chemokine signaling in DRG neurons and connective tissue mast cells is given.

Up-regulation of CCR2 in the process of pain chronification is often associated with increased release of its endogenous agonist CCL2 in DRG neurons or immune cells. As shown in figure 4A, BAM8-22 did not induce CCL2 expression in F11-MRGPR-X1 cells, excluding autocrine F11 cell activation via CCL2. Cp have been reported to be associated with DRG neurons and to produce CCL2, leading to paracrine stimulation of CCR2 expressing DRG neurons [60]. Herein, we provide first functional data, indicating that MRGPR-X1 are expressed in the CTMC-derived LAD-2 cell line and thus, exert their action in cells of the peripheral nervous and the immune system. However, so far no data about the physiological role of MRGPR-X1-induced signaling in mast cells are available. Interestingly, we found BAM8-22-promoted CCL2 release in LAD-2 cells and thus, describe the first putatively biological relevant effect of MRGPR-X1 in mast cells. Furthermore our data suggest that MRGPR-X1 may induce a chemokine signaling circuit involving two different mechanisms in distinct, yet functionally co-operating cell types (see figure 8). As mentioned earlier, MRGPR-X1 belong to the small group of GPCR that do not undergo agonist-promoted desensitisation or internalisation [6]. Thus, the predicted effects of BAM8-22 on the chemokine signaling circuit would not be diminished by cellular mechanisms that usually down-regulate GPCR signaling. Hence, one may postulate that prolonged MRGPR-X1-promoted signaling may have sustained effects on CCR2/CCL2 signaling and thus, on chemokine-induced pain chronification in humans.

### Pitfalls and Perspectives

Rodents play a central role as *in vivo* models for pain research. Primate-specific expression is therefore the major limitation in the work with human MRGPR-X1, as no ortholog rodent *in vivo* models exist. Of note, there is one receptor subtype in rodents, named mas-related G protein-coupled receptor-C (MRGPR-C) that is also expressed in DRG neurons, activated by BAM8-22 and shares ∼50% sequence homology with MRGPR-X1. In addition to BAM8-22, murine MRGPR-C are activated by γ_2_-melanocyte stimulating hormone (γ_2_-MSH), dynorphin-14 and neuropeptides NPFF or NPAF [61], [62]. Overall it is difficult to determine whether rodent MRGPR-C are suitable MRGPR-X1 counterparts. Thus, it has been under debate whether the function of human MRGPR-X1 can be accurately extrapolated from studies with rodent MRGPR-C [7], [10]. To obtain more information about the functional characteristics of different MRGPR, we determined the ligand profile of human MRGPR-X1, rat and mouse MRGPR-C in HEK293 cells stably expressing either receptor [6], by monitoring changes in intracellular calcium levels induced by various concentrations of all peptides reported to activate the murine MRGPR-C (Supp. Fig. S1). Thereby we observed that BAM8-22, γ_2_-MSH and dynorphin-14 are full agonists and NPFF/NFAF partial agonists of the murine MRGPR-C. Surprisingly, rat MRGPR-C were activated only by γ_2_-MSH and BAM8-22, with γ_2_-MSH being more potent than BAM8-22, but not by dynorphin-14 or NPFF/NFAF, suggesting differences in the ligand profile between mice and rats. Of all tested peptides, BAM8-22 was the only one that induced significant calcium signals in MRGPR-X1 expressing cells. Thus, these experiments clearly indicate striking differences in the ligand profile between rodent and human MRGPR. Interestingly, differences in the pharmacodynamics of GPCR from distinct species are not restricted to MRGPR but have also been observed for other GPCR such as histamine receptors [63], free fatty acid receptors 2 and 3 [64], G-protein-coupled receptor 35 [65], trace amine-associated receptors 1 [66], kappa opioid receptors [67] and beta-adrenergic receptors [68] and thus, might be an underestimated problem for the development of GPCR-targeting drugs. However, in conjunction with fundamental differences in ligand-promoted desensitization [6] and the lack of MRGPR-C expression in murine mast cells [3], we reasoned that rodents would not qualify as suitable model systems for our study. Thus, our results obtained at the cellular level still have to await final physiological proof.

Our data define MRGPR-X1 as modulators of gene expression and thereby as inducers of chronic pain markers such CCR2. Furthermore, we identified MRGPR-X1 as positive regulators of CCL2 release in mast cells. Thus, MRGPR-X1 may not only be therapeutic targets for the treatment of acute, but also for chronic pain. Given their exclusive expression in DRG neurons and mast cells, MRGPR-X1-based pain therapy would selectively affect cell types that are pertinent to pain chronification, thereby minimizing the risk of side effects. To validate this hypothesis in the future, it is imperative to establish primate-based, DRG neuron-derived model systems. Furthermore, testing recently described MRGPR-X1 specific antagonists in humans suffering from neuropathic pain may represent an invaluable approach to delineate the pathophysiological role of MRGPR-X1 signaling in human pain chronification [69].

## Supporting Information

Figure S1 **Distinct ligand profile of human MRGPR-X1 and rodent MRGPR-C.** Calcium concentration-response curves in fura2-loaded stably MRGPR-X1 (A), murine MRGPR-C (B) or rat MRGPR-C (C) expressing cells and in coelenterazine H-loaded HEK293-Ae cells stably expressing MRGPR-X1 (D), murine MRGPR-C (E) or rat MRGPR-C (F) were monitored, after injection of various concentrations of BAM8-22 (blue), γ_2_-MSH (orange), dynorphin-14 (green), dynorphin-A (black), neuropeptide FF (brown) or neuropeptide AF (red). Background signals induced by injection of HBS were subtracted and responses normalized by defining the calcium transients elicited by the full agonist as 100%. Results are expressed as the mean ± S.E.M. of at least 4 independent experiments performed in duplicates. Curve fittings were carried out using the sigmoid dose-response (variable slope) algorithm of Prism4.0.
(TIF)
