# Supplementary figures and images for: Human Mas-Related G Protein-Coupled Receptors-X1 Induce Chemokine Receptor 2 Expression in Rat Dorsal Root Ganglia Neurons and Release of Chemokine Ligand 2 from the Human LAD-2 Mast Cell Line
Source: PLoS One. 2013 Mar 7;8(3):e58756. doi: 10.1371/journal.pone.0058756 (PMC3591377; doi:10.1371/journal.pone.0058756)

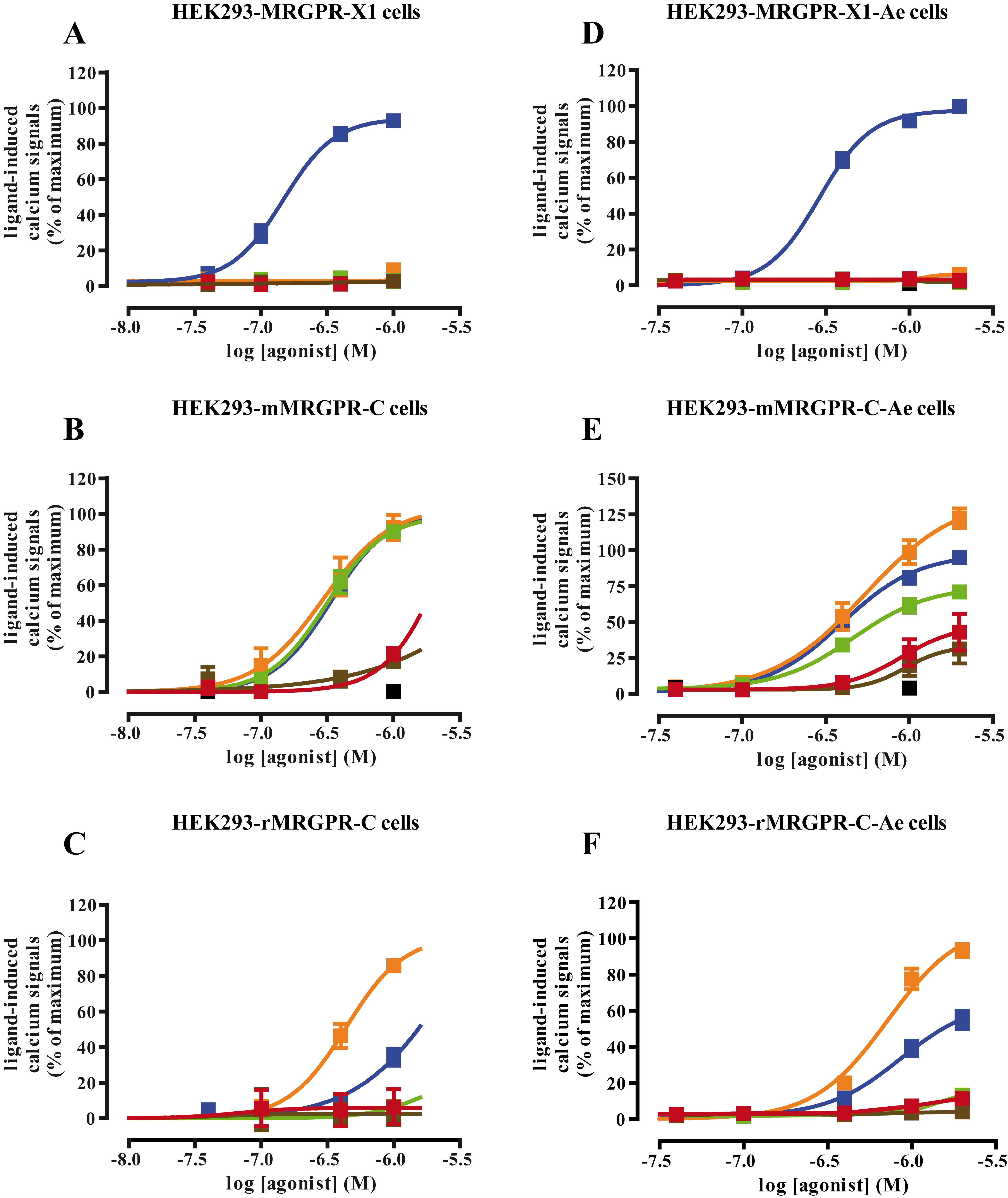

Supplement: Figure S1 — Distinct ligand profile of human MRGPR-X1 and rodent MRGPR-C. Calcium concentration-response curves in fura2-loaded stably MRGPR-X1 (A), murine MRGPR-C (B) or rat MRGPR-C (C) expressing cells and in coelenterazine H-loaded HEK293-Ae cells stably expressing MRGPR-X1 (D), murine MRGPR-C (E) or rat MRGPR-C (F) were monitored, after injection of various concentrations of BAM8-22 (blue), γ2-MSH (orange), dynorphin-14 (green), dynorphin-A (black), neuropeptide FF (brown) or neuropeptide AF (red). Background signals induced by injection of HBS were subtracted and responses normalized by defining the calcium transients elicited by the full agonist as 100%. Results are expressed as the mean ± S.E.M. of at least 4 independent experiments performed in duplicates. Curve fittings were carried out using the sigmoid dose-response (variable slope) algorithm of Prism4.0. (TIF) [file pone.0058756.s001.tif]
